# Frequency and Factors Associated with Adult Immunization in Patients Visiting Family Medicine Clinics at a Tertiary Care Hospital, Karachi

**DOI:** 10.7759/cureus.2083

**Published:** 2018-01-17

**Authors:** Samar Zaki, Asma Usman, Swaleha Tariq, Sameena Shah, Iqbal Azam, Waris Qidwai, Kashmira Nanji

**Affiliations:** 1 Family Medicine, The Aga Khan University; 2 Community Health Sciences, The Aga University

**Keywords:** immunization, patients, vaccines, awareness

## Abstract

Objective

The goal of this study was to determine the frequency and factors associated with adult immunization in patients visiting family medicine clinics at a tertiary care hospital in Karachi.

Methods

A cross-sectional study was conducted from March 2014 to March 2015 in a tertiary care hospital in Karachi, Pakistan. Participants more than 18 years were invited to participate in the study. A pretested questionnaire was used to collect information. Data were entered and analyzed using IBM SPSS Statistics for Windows, version 19.0 (Armonk, NY: IBM Corp).

Results

A total of 340 patients were surveyed. The majority of patients were female (69.5%) with a mean age of 35.47 years. The majority were married (61.1%), and 30% of the participants had completed graduation or postgraduate education (20%). Most of the patients believed that vaccines can be used in adults to prevent disease (62.2%). Patients believed that the hepatitis B vaccine, influenza vaccine, and hepatitis A vaccine can be administered to adults (58.1%, 29.9%, 33.8%, respectively). The major sources of their information regarding vaccination in adults were friends or relatives (25%) and media (23.2%). Regarding availability of vaccines, 71.3% thought a hepatitis B vaccine is available, 54.9% thought a tuberculosis vaccine is available, and 49.3% thought a tetanus toxoid vaccine is available. Only 36.4% respondents received any vaccine in adulthood. The majority of patients (62.2%) received the hepatitis B vaccine in adulthood. The major reason given for not receiving vaccines was lack of awareness (62.4%).

Conclusion

Low adult vaccination coverage rates and awareness, as highlighted by the results of this study, show the dire need to address this major preventive strategy. This information can be utilized to conduct larger community-based surveys, to conduct health awareness sessions in the community, and to educate our doctors regarding the availability and benefits of adult vaccines.

## Introduction

Immunization is the process whereby a person is made immune or resistant to an infectious disease typically by administration of a vaccine [1]. It is a safe, effective, and simple way to prevent life-threatening illnesses not only in children but also in adults. Vaccines are some of the safest medicines available that can relieve suffering costs related to these preventable diseases [1].

According to Centers for Disease Control and Prevention (CDC), there is a strong need for vaccinations from birth through adulthood to provide lifetime immunity [1,2]. Adults are more likely to die from vaccine-preventable diseases than children [1,2]. Most adults fail to receive any recommended vaccines, thus leaving them needlessly vulnerable to illness, long-term suffering, and even death. Vaccination status worldwide, as cited in the CDC national immunization survey report, has shown some improvement but is still below the target rate. According to one estimate, 50,000 adults in the United States (US) die every year secondary to vaccine-preventable diseases [3,4].

An international survey conducted in the US reported that only 31% of family physicians assessed adult patients for hepatitis B risk factors and advised vaccination to patients identified as high-risk [5]. The vaccination rate for tetanus and chicken pox has improved, resulting in 51.5% and 6.7% coverage, respectively, whereas no change in human papillomavirus vaccine status has been reported. Similarly, very little improvement has been seen in pneumococcal and influenza vaccine coverage rates [5-8].

Immunization coverage in Pakistan is still low and needs improvement [9]. There are various barriers including fear of side effects, mistaken beliefs about vaccine mode of action, high cost, fear of needles, and rumors regarding questionable vaccine efficacy [10-15]. Of the factors mentioned above, lack of awareness among the general population is an important area of concern. A survey done in family medicine clinics in a tertiary care hospital showed very little knowledge; 24% of the patients presenting to the clinic lacked awareness of adult immunizations despite a high level of educational attainment [11]. Lack of awareness among physicians is also a significant factor leading to low rates of adult immunization. A survey done in 2007 showed that the majority of patients (79% to 85%) said that they would get vaccinated if their doctor recommended them, but only 41% said that they would get vaccinated without the recommendation [11].

The current healthcare system in Pakistan focuses on acute treatment and is poorly equipped to deliver preventive medicine to the population, especially to adults [10]. Access to preventive services, when they are offered, is limited, and neither the government nor the private sector has been able to develop a sustained adult vaccine infrastructure, and this itself is an important hurdle in adult vaccination [10, 11].

Although numerous researches have been conducted on childhood immunization and incorporated into the Extended Program of Immunization (EPI), limited data exist regarding adult immunization and its factors, especially in developing countries. This study will elucidate the frequency and factors of adult immunization from patients’ perspectives in Pakistan.

## Materials and methods

We conducted a cross-sectional study from March 2014 to March 2015 in the Community Health Centre (a group of family medicine clinics), at a tertiary hospital, Aga Khan University Hospital (AKUH), in Karachi, Pakistan. Twelve family physicians see over approximately 150 patients of different socioeconomic backgrounds at this center during a typical day.

The sample size was calculated using the STEPS Sample Size Calculator from the World Health Organization. To determine the frequency of immunization among adults visiting all family medicine clinics affiliated with AKUH, the prevalence of adult immunization was assumed to be 26%. We estimated with 95% confidence and 10% error the sample size to be 289. After adding 5% for non-response, the final sample size was calculated to be 339 study participants.

A total of 340 adult patients aged 19 years and above were recruited from Community Health Centre after obtaining informed written consent. Patients with hearing problems, mental disability, or with language barrier were excluded. The study was approved by the ethical review committee of Aga Khan University. The nature and purpose of the study were explained in detail to the participants. A pre-tested, structured questionnaire was used to collect information. The questionnaire was divided into two components. The first section included questions related to personal demographic profile, such as age, education, marital status, employment, and household income. The second section assessed patient knowledge regarding the status of adult immunization and its associated factors. The questionnaire was initially developed in English, translated to Urdu, and back-translated to English. The principal investigator conducted a brief training session for interviewers to ensure uniform data collection. A pilot study was performed in 5% of the calculated sample size.

Data were entered in IBM SPSS Statistics for windows, version 19.0 (Armonk, NY: IBM Corp). Frequencies for categorical variables such as gender and marital status, mean and standard deviation of continuous variables such as age, and frequencies of all the vaccinations received in adulthood were calculated. The Chi-square test was used to find an association of knowledge with demographic variables. All analyses were done with two-tailed tests, and p < 0.05 was considered statistically significant.

## Results

A total of 340 patients were surveyed. The majority were female (69.5%) with a mean age of 35.47 years. The majority were married (61.1%), and about 30% of the participants had completed graduation or postgraduate education (20%). The majority owned a house (67.8%), and household size was three to four rooms (51.6%) with seven or more persons living per house (40.5%) (Table 1).

**Table 1:** Demographic profile of respondents.

Parameter	Number (%)
Age (years)	
<25	94 (25.4)
25-34	105 (28.4)
35-49	98 (26.5)
50-59	41 (11.1)
>60	32 (8.6)
Mean age in years (± standard deviation)	35.47 (± 14.44)
Gender	
Male	113 (30.5)
Female	257 (69.5)
Marital status	
Single	123 (33.2)
Married	226 (61.1)
Divorced/widowed	21 (5.7)
Education	
Illiterate	48 (13)
Below matric	42 (11.4)
Matric	35 (9.5)
Intermediate	59 (15.9)
Graduate	111 (30)
Postgraduate	75 (20.3)
Ownership of house	
Owned	251 (67.8)
Rented	110 (29.7)
Household size (number of rooms)	
≥2	101 (27.3)
3-4	191 (51.6)
5-6	56 (15.1)
>7	22 (5.9)
Household size (number of persons)	
≥2	26 (7)
2-4	82 (22.2)
5-6	112 (30.3)
>7	150 (40.5)

A majority of the participants believed that vaccines can be used in adults to prevent disease (62.2%). Patients believed that the hepatitis B vaccine, influenza vaccine, and hepatitis A vaccine can be administered to adults (58.1%, 29.9%, 33.8%, respectively). The major source of their information regarding vaccination in adults was friends/relatives (25%) and media (23.2%). Only 10% of the respondents received information from doctors. Regarding availability of vaccines, 71.3% thought a hepatitis B vaccine is available, 54.9% thought a tuberculosis vaccine is available, and 49.3% thought a tetanus toxoid vaccine is available. Similarly, 47.1% thought that the hepatitis C vaccine is available, and 28.3% thought that malaria vaccine is available.

Only 36.4% respondents received any vaccine in their adulthood. The majority (62.2%) received the hepatitis B vaccine in adulthood. The major reason given for not receiving vaccines was lack of awareness (62.4%). Nearly all patients (94.2%) were in favor of education on immunization. The respondents (79.8%) believed that media is a good source to promote adult immunization. Most respondents (93.9%) would recommend immunization to other adults (Table 2).

**Table 2:** Responses of study subjects regarding immunization.

Question	Number (%)
Which of the following vaccines can be given in adults?	
Tetanus toxoid	126 (34.1)
Hepatitis B	215 (58.1)
Measles/measles, mumps, rubella (MMR)	62 (16.8)
Influenza	110 (29.9)
Pneumococcal	50 (13.5)
Varicella	44 (11.9)
Typhoid	103 (27.8)
Hepatitis A	125 (33.8)
Meningitis	60 (16.2)
Human papillomavirus	39 (10.5)
Which of the following vaccines are available?	
Tetanus toxoid	176 (49.3)
Hepatitis B	254 (71.3)
MMR	113 (31.7)
Influenza	136 (38.1)
Malaria	101 (28.3)
Pneumococcal	81 (22.8)
Varicella	71 (19.9)
Typhoid	150 (42.1)
Hepatitis C	168 (47.1)
Hepatitis A	162 (45.4)
Meningitis	83 (23.2)
Human papillomavirus	195 (54.9)
Have you ever received any of the following vaccines in adulthood?	
Tetanus toxoid	44 (29.7)
Hepatitis B	92 (62.2)
Measles/MMR	17 (11.5)
Influenza	11 (7.5)
Pneumococcal	2 (1.4)
Varicella	2 (1.4)
Typhoid	7 (4.8)
Hepatitis A	7 (4.8)
Meningitis	6 (4.1)
Human papillomavirus	1 (0.7)
Source of information about vaccines	
Friends/relatives	93 (25.1)
Doctors	39 (10.5)
Media/newspaper/internet	86 (23.2)
Reasons of not receiving vaccines?	
Lack of awareness	154 (62.4)
Didn’t think it’s needed	81 (33.1)
Has harmful effects	88 (23.7)
Costly	28 (11.4)
Doctor didn’t advise	53 (21.6)
How adult immunization can be promoted?	
General practitioner	79 (22.1)
Specialist	34 (9.5)
Media	285 (79.8)
Newspaper	108 (30.3)
Internet	80 (22.5)
Can vaccines be used in adults to prevent disease?	
Yes	230 (62.2)
No	140 (37.8)
Have you ever received any vaccine in adulthood?	
Yes	133 (36.4)
No	220 (60.3)
Would you like an awareness session about immunization?	
Yes	344 (94.2)
No	18 (4.9)
Would you recommend immunization to adults?	
Yes	337 (93.9)
No	12 (3.3)

A comparison of vaccine knowledge was made between the demographic variables. When asked if vaccines can be given to adults to prevent diseases, 77% of the respondents younger than 25 years knew that vaccines can be given to adults. Among male and female respondents, 57.5% and 64.2% knew that vaccines can be given in adults to prevent diseases, respectively. Among single respondents, 74% knew about adult vaccines, and 57.5% of married respondents knew about adult vaccines. The majority of respondents (82.7%) with a postgraduate education knew about adult vaccines, whereas 75% of uneducated respondents did not know that adult vaccines can prevent diseases. Approximately 63% of those who owned a house vs 57.3% of those who were living in rented houses knew that adult vaccines can prevent diseases (Table 3).

**Table 3:** Association of demographic variables with knowledge of adult vaccines to prevent disease.

Variables	Knowledge of vaccines to prevent disease Number (%)	p-value
Age (years)		
<25	73 (77)	0.000
25-34	68 (64.8)	
35-49	53 (54.1)	
50-59	17 (41.5)	
>60	19 (59.4)	
Gender		0.222
Male	65 (57.5)	
Female	165 (64.2)	
Marital status		
Single	91 (74)	0.002
Married	130 (57.5)	
Divorced/widowed	9 (42.9)	
Education status		
Illiterate	12 (25)	0.000
Below matric	14 (33.3)	
Matric	18 (51.4)	
Intermediate	45 (76.3)	
Graduate	79 (71.2)	
Postgraduate	62 (82.7)	
Socioeconomic status		
Owned house	159 (63.3)	0.135
Rented house	63 (57.3)	

## Discussion

This survey provides an understanding of the knowledge, attitude, and practices regarding immunization among patients presenting in the family medicine clinics. The majority of the respondents were females and were married. This was in concordance with other studies, which have shown that women have a higher mean number of visits to their primary care clinics than men [1].

The majority of the respondents (62%) believed that immunization is protective against diseases. This was concordant with similar studies done previously where the majority believed that vaccination prevents disease [11,16].

The knowledge regarding different vaccines was variable, with the hepatitis B vaccination being the most familiar followed by influenza and hepatitis A. A study on hepatitis B in Quetta, Pakistan showed that 77% of participants had knowledge about the hepatitis B vaccine [17]. This can be explained by the fact that awareness regarding hepatitis B vaccination is widespread because of media and newspaper coverage and through family/friends. Our study also showed that knowledge regarding vaccination was greater in people with higher education. This is similar to another study which showed that higher education levels were associated with higher vaccination rates [18]. Another interesting finding was that the vaccination knowledge was also high in people with higher socioeconomic status. Studies from Spain and the US have also shown that patients with higher income had significantly higher immunization rates [19,20].

Another interesting but disappointing fact was that only 10% received immunization information from their doctors. This could be the result of doctors’ lack of time or knowledge. One study from India showed that doctors’ knowledge regarding immunization was deficient [20]. A study done on pneumococcal vaccination rates in people over 65 years from Pittsburgh, Pennsylvania, in the US revealed that a recommendation by a physician or friend or relative was associated with significantly higher pneumococcal vaccination rates (p < 0.001) [21].

Our study indicates that about 36.4% of the participants receive vaccination in adulthood. Data from the National Health Interview Survey in the US showed vaccination coverage rates of tetanus, diphtheria, and pertussis among adults 19 years and older were 17.2% in 2014; herpes zoster vaccination among adults 60 years and older coverage rates were 24.2% [22].

A Canadian Adult National Immunization Coverage Survey in 2014 revealed that less than half of the adults (40%) received a dose of the influenza vaccine for the 2013-2014 season [23]. Approximately 50% of Canadian adults reported receiving a vaccine against tetanus in the past 10 years, 37% of adults reported having received at least one dose of the pneumococcal vaccine in their lifetime, and 72% of healthcare providers received hepatitis B vaccination [23]. A 2015 study of 1350 Iranian adults 60 years or older showed that only 10% had received influenza vaccinations within the last year [24].

These low adult vaccination rates, including from developed countries, is quite distressing and shows that it is a major public health problem across the globe.

Our study had limitations. We used convenience sampling, which limits the external validity of the study. The information was acquired via a face-to-face interview and based on a questionnaire. While interviewer encouragement may have led to higher rates of completion, it may also have introduced the interviewer's bias in data collection. However, the questionnaire was thoroughly discussed with the interviewer before data collection to reduce interview bias. Also, the data were collected from one hospital; therefore, we cannot generalize it to the entire adult population.

## Conclusions

This study provides good insight into the knowledge, attitude, and practices of adult immunization among patients visiting family clinics. This information can be utilized to conduct larger community-based surveys. The media and family and friends proved to be important to spreading knowledge. We need to utilize this important source of information and conduct health awareness sessions in the community so that information is widely conveyed regarding the benefits of adult immunization. We also need to educate doctors regarding the availability and benefits of adult vaccines. Low adult vaccination coverage rates and awareness worldwide shows the dire need to address this major preventive strategy at both national and international level.
